# Randomized Cross-Over Trial of Electrolyte, Acid-Base and Blood Pressure Effects of Salt Supplements in CKD

**DOI:** 10.1016/j.ekir.2026.106619

**Published:** 2026-05-26

**Authors:** Michiel L.A.J. Wieërs, Usha Musterd-Bhaggoe, Yoëlle Goos, Ingrid M. Garrelds, Sjoerd A.A. van den Berg, A.H. Jan Danser, Lynda A. Frassetto, Martin H. de Borst, Liffert Vogt, Joris I. Rotmans, Pedro H. Imenez Silva, Ewout J. Hoorn

**Affiliations:** 1 Division of Nephrology and Transplantation, Department of Internal Medicine, Erasmus Medical Center, University Medical Center Rotterdam, Rotterdam, The Netherlands; 2 Division of Pharmacology, Department of Internal Medicine, Vascular Medicine and Metabolic Diseases Erasmus Medical Center, University Medical Center Rotterdam, Rotterdam, The Netherlands; 3 Department of Clinical Chemistry, Erasmus Medical Center, University Medical Center Rotterdam, Rotterdam, The Netherlands; 4 Division of Nephrology, Department of Medicine, University of California San Francisco, San Francisco, California, USA; 5 Division of Nephrology, Department of Internal Medicine, University Medical Center Groningen, Groningen, The Netherlands; 6 Division of Nephrology, Department of Internal Medicine, Amsterdam University Medical Centers, Amsterdam Cardiovascular Sciences, Amsterdam, The Netherlands; 7 Division of Nephrology, of Internal Medicine, Leiden University Medical Center, Leiden, The Netherlands

## Introduction

In individuals with normal kidney function, increasing potassium intake reduces blood pressure irrespective of the accompanying anion and with minimal effects on electrolyte and acid-base parameters.^1^, ^2^, ^3^ However, the effects of potassium and its accompanying anion in patients with chronic kidney disease (CKD) are unclear and raise concerns regarding hyperkalemia. Our previous analysis showed that 40 mmol/d of potassium chloride supplementation increased plasma potassium by 0.4 mmol/l and caused mild metabolic acidosis, which could counteract the beneficial effects of potassium.^4^^,^^5^ These findings highlight the need for a head-to-head comparison of salt supplements with varying compositions in patients with CKD to assess their effects on electrolyte balance, acid-base status, and blood pressure, which is the objective of the present study.

## Results

### Patient Characteristics

A total of 283 patients were screened, 72 were eligible, and 31 agreed to participate (Supplementary Figure S1). Thirty patients completed all 6 treatment periods, and 1 patient discontinued the trial after 3 periods due to time demands. The 31 patients included in the study were aged a mean of 69 ± 11 years with an estimated glomerular filtration rate of 29 ± 7 ml/min per 1.73 m^2^, 29% were female, and 39% had type 2 diabetes (Supplementary Table S1).

### Acid-Base Balance

**Figure 1:**
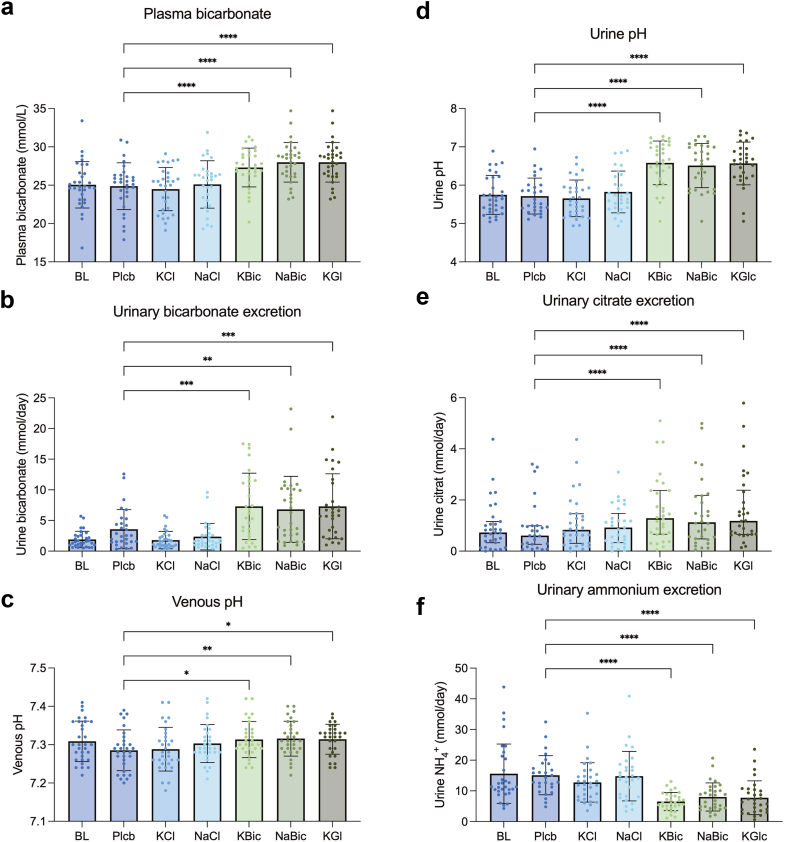
Effects of supplements on acid-base parameters in blood and urine. Data were analyzed using a linear mixed-effects model with subject included as a random intercept and treatment and period included as fixed effects. Post-hoc comparisons were adjusted for multiple testing using Tukey’s test. BL, baseline; KBic, potassium bicarbonate; KCl, potassium chloride; KGlc, potassium gluconate; NaBic, sodium bicarbonate; NaCl, sodium chloride; Plcb, placebo. Adjusted *P*-values: ∗*P* ≤ 0.05, ∗∗*P* ≤ 0.01, ∗∗∗*P* ≤ 0.001. ∗∗∗∗*P* ≤ 0.0001.

Compared with placebo, potassium chloride caused a nonsignificant decrease in plasma bicarbonate (−0.4 mmol/l), whereas potassium bicarbonate, sodium bicarbonate, and potassium gluconate significantly increased plasma bicarbonate (mean: 27–28 vs. 25 mmol/l, Figure 1a). Urinary bicarbonate excretion was significantly higher with these supplements (mean: 6 vs. 3 mmol/d; Figure 1b). The alkalizing effects were further reflected in significantly higher venous and urinary pH values (Figure 1c and d), increased urinary citrate excretion (Figure 1e), reduced urinary ammonium excretion (Figure 1f) and reduced net acid excretion (Supplementary Figure S2).

### Electrolytes

**Figure 2:**
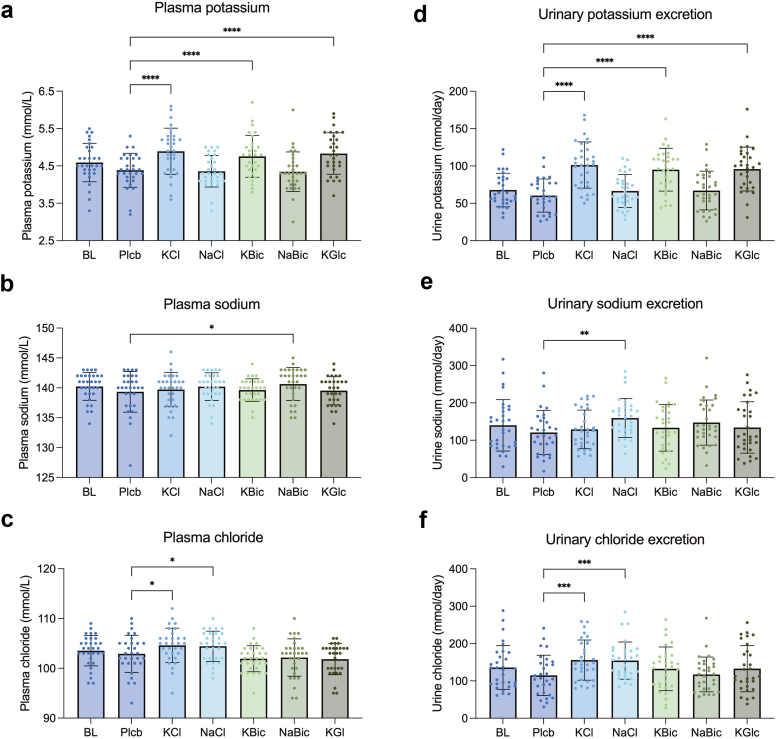
Effects of supplements on plasma levels and urinary excretion of potassium, sodium, and chloride. Data were analyzed using a linear mixed-effects model with subject included as a random intercept and treatment and period included as fixed effects. Post-hoc comparisons were adjusted for multiple testing using Tukey’s test. BL, baseline; KBic, potassium bicarbonate; KCl, potassium chloride; KGlc, potassium gluconate; NaBic, sodium bicarbonate; NaCl, sodium chloride; Plcb, placebo. Adjusted *P*-values: ∗*P* ≤ 0.05, ∗∗*P* ≤ 0.01, ∗∗∗*P* ≤ 0.001, ∗∗∗∗*P* ≤ 0.0001.

Compared with placebo, plasma potassium levels were significantly higher during treatment with all 3 potassium salts (mean: 4.8–4.9 vs. 4.4 mmol/l; Figure 2a). Six patients developed hyperkalemia while receiving one of the potassium supplements (plasma potassium range: 5.6–6.2 mmol/l; Supplementary Table S2). In these patients, baseline plasma potassium was significantly higher, and estimated glomerular filtration rate was significantly lower (Supplementary Table S3). In all 6 patients, plasma potassium normalized after washout, allowing them to continue with the study (Supplementary Figure S3). Plasma sodium levels were significantly higher during treatment with sodium bicarbonate (mean: 141 vs. 139 mmollL; Figure 2b), whereas plasma chloride levels were significantly higher during treatment with both potassium chloride and sodium chloride (mean: 104–105 vs. 103 mmol/l; Figure 2c). Treatment adherence was confirmed by increased urinary potassium, sodium, and chloride excretions (Figure 2d–f) , although urinary sodium excretion did not significantly increase with sodium bicarbonate. No significant differences in the effects of the supplements on plasma bicarbonate or potassium were observed when comparing thiazide versus nonthiazide users (Supplementary Table S4).

### Blood Pressure, Plasma Renin, and Aldosterone

Average 4-day home systolic blood pressure and mean arterial pressure were significantly higher during treatment with potassium and sodium chloride compared with placebo, whereas no effect on diastolic blood pressure was observed (Supplementary Figure S4a–c). Systolic blood pressure during treatment with potassium gluconate was significantly lower than during treatment with potassium chloride or sodium bicarbonate. Plasma renin levels were significantly lower during treatment with sodium chloride (Supplementary Figure S4d). No significant differences in plasma aldosterone were observed between placebo and any of the treatments (Supplementary Figure 4e). No differences in body weight, estimated glomerular filtration rate and albuminuria were observed between placebo and any of the treatments (Supplementary Figure S5).

### Plasma Magnesium, Phosphate, Endothelin-1, and Urine Cortisol

No significant differences in plasma magnesium, calcium, or phosphate were observed between placebo and any of the treatments (Supplementary Figure S6). Plasma endothelin-1 and the urine cortisol-to-cortisone ratio were measured because of their reported roles in acid-base balance regulation^6^^,^^7^; however, no differences were observed across treatments (Supplementary Figure S7).

### Safety

Except for hyperkalemia, no (serious) adverse events occurred and none of the patients required additional antihypertensive medication during treatment with any of the supplements.

## Discussion

In patients with CKD, potassium supplements raise plasma potassium regardless of the accompanying anion or acid–base changes; potassium gluconate shows bicarbonate-like alkalizing effects, and potassium chloride uniquely increases blood pressure whereas bicarbonate and gluconate do not.

The effects of potassium supplements in patients with CKD clearly differ from those previously observed in patients with hypertension.^1^, ^2^, ^3^ Our results are consistent with the BASE pilot trial, in which the higher-dose sodium bicarbonate—comparable with our dose when adjusted for lean body weight—increased plasma bicarbonate from 24 to 26 mmol/l and reduced urinary ammonium excretion by approximately 50%.^8^ Previous studies found that a higher dietary acid load increased urinary glucocorticoids and that oral bicarbonate reduced plasma endothelin-1^6^^,^^7^; we did not observe effects on these parameters in our study.

The alkalizing effect of gluconate was unexpected, because it is generally considered to have little or no effect on acid-base balance.^9^^,^ S1 One previous study found that 85 mmol/d of potassium gluconate in prehypertensive individuals increased urine pH by 0.46.S2 In our study, the effect was clearly stronger: 40 mmol/d increased urine pH by 1.1. Bicarbonate salts can cause gastrointestinal side effects due to carbon dioxide release when reacting with stomach acid,S3 making gluconate-containing salts a suitable alternative for alkaline therapy.

The effect of potassium supplements on plasma potassium in this study was similar to that of our previous study with potassium chloride.^4^ In contrast, potassium chloride supplementation here did not have a significant acidifying effect and increased blood pressure. The 3 potassium salts increased urinary potassium excretion from approximately 60 to 100 mmol/d, bringing dietary potassium intake within the recommended range.S4^,^S5 The blood pressure effects of the potassium salts suggest that CKD and/or the use of renin-angiotensin inhibitors may shift the potassium intake–blood pressure curve leftward.S4 In addition, potassium-induced natriuresis is impaired in CKD, which may affect the blood pressure response to potassium.S6 The hypertensive effect of potassium chloride has been characterized in rodent studies, with potential roles for chloride and aldosterone.S7–S9

The strength of this study is its randomized, double-blind, placebo-controlled design. Limitations include an uncontrolled diet despite the recommendation to maintain stable intake across periods, potential differences between acute and chronic responses to increased dietary potassium intake,S10 and limited generalizability of supplements versus potassium-rich diets. In addition, the washout period and time to reach steady state were relatively short, although consistent with previous randomized crossover trials.S11^,^S12

In conclusion, in patients with CKD, chloride salts increase plasma chloride and blood pressure, whereas potassium salts increase plasma potassium irrespective of acid-base balance. This response differs from individuals with normal kidney function and implies that specific strategies are needed to guide the efficacy and safety of adequate potassium intake in patients with CKD.

## Disclosure

MHdB reported grants from CSL Vifor and consulting fees from Astellas, AstraZeneca, Bayer, Boehringer Ingelheim, CSL Vifor, Kyowa Kirin Pharma, Lilly, and Sanofi Genzyme (all paid to institution). JIR reports grants from AstraZeneca (paid to institution). EJH reports royalties from UpToDate. All the other authors declared no competing interests.

## Supplementary Material

Supplementary File (PDF) **Supplementary Methods. Supplementary References. Figure S1.** CONSORT diagram. **Figure S2.** Effects of salt supplements on net acid excretion. **Figure S3.** Flowchart for management of hyperkalemia. **Figure S4.** Effects of supplements on home systolic blood pressure, diastolic blood pressure, mean arterial pressure, plasma renin and aldosterone. **Figure S5.** Effects of salt supplements on body weight, eGFR, and albuminuria. **Figure S6.** Effects of salt supplements on plasma magnesium, calcium, and phosphate. **Figure S7.** Effects of salt supplements on endothelin-1 and urine cortisol to cortisone ratio. **Table S1.** Baseline characteristics. **Table S2.** Characteristics of patients developing hyperkalemia. **Table S3.** Comparison of baseline characteristics between patients who did or did not develop hyperkalemia during one of the treatments. **Table S4.** Subanalysis comparing plasma bicarbonate and potassium in participants with or without thiazide diuretic use. CONSORT Checklist.

## Footnotes

Supplementary File (PDF)

**Supplementary Methods.**

**Supplementary References.**
**Figure S1.** CONSORT diagram. **Figure S2.** Effects of salt supplements on net acid excretion. **Figure S3.** Flowchart for management of hyperkalemia. **Figure S4.** Effects of supplements on home systolic blood pressure, diastolic blood pressure, mean arterial pressure, plasma renin and aldosterone. **Figure S5.** Effects of salt supplements on body weight, eGFR, and albuminuria. **Figure S6.** Effects of salt supplements on plasma magnesium, calcium, and phosphate. **Figure S7.** Effects of salt supplements on endothelin-1 and urine cortisol to cortisone ratio. **Table S1.** Baseline characteristics. **Table S2.** Characteristics of patients developing hyperkalemia. **Table S3.** Comparison of baseline characteristics between patients who did or did not develop hyperkalemia during one of the treatments. **Table S4.** Subanalysis comparing plasma bicarbonate and potassium in participants with or without thiazide diuretic use. CONSORT Checklist.

## References

[1] Braschi A., Naismith D.J. (2008). The effect of a dietary supplement of potassium chloride or potassium citrate on blood pressure in predominantly normotensive volunteers. Br J Nutr.

[2] He F.J., Marciniak M., Carney C. (2010). Effects of potassium chloride and potassium bicarbonate on endothelial function, cardiovascular risk factors, and bone turnover in mild hypertensives. Hypertension.

[3] He F.J., Markandu N.D., Coltart R., Barron J., MacGregor G.A. (2005). Effect of short-term supplementation of potassium chloride and potassium citrate on blood pressure in hypertensives. Hypertension.

[4] Gritter M., Wouda R.D., Yeung S.M.H. (2022). Effects of short-term potassium chloride supplementation in patients with CKD. J Am Soc Nephrol.

[5] Toto R.D., Hulter H.N., Mackie S., Sebastian A. (1984). Renal tubular acidosis induced by dietary chloride. Kidney Int.

[6] Esche J., Shi L., Sanchez-Guijo A., Hartmann M.F., Wudy S.A., Remer T. (2016). Higher diet-dependent renal acid load associates with higher glucocorticoid secretion and potentially bioactive free glucocorticoids in healthy children. Kidney Int.

[7] Wesson D.E., Simoni J., Broglio K., Sheather S. (2011). Acid retention accompanies reduced GFR in humans and increases plasma levels of endothelin and aldosterone. Am J Physiol Ren Physiol.

[8] Raphael K.L., Isakova T., Ix J.H. (2020). A randomized trial comparing the safety, adherence, and pharmacodynamics profiles of two doses of sodium bicarbonate in CKD: the BASE pilot trial. J Am Soc Nephrol.

[9] Kirkendol P.L., Starrs J., Gonzalez F.M. (1980). The effects of acetate, lactate, succinate and gluconate on plasma pH and electrolytes in dogs. Trans Am Soc Artif Intern Organs.

