# Supplementary material for: Randomized Cross-Over Trial of Electrolyte, Acid-Base and Blood Pressure Effects of Salt Supplements in CKD
Source: Kidney Int Rep. 2026 May 26;11(8):106619. doi: 10.1016/j.ekir.2026.106619 (PMC13315686; doi:10.1016/j.ekir.2026.106619)
Supplement: Supplementary File (PDF) — Supplementary Methods. Supplementary References. Figure S1. CONSORT diagram. Figure S2. Effects of salt supplements on net acid excretion. Figure S3. Flowchart for management of hyperkalemia. Figure S4. Effects of supplements on home systolic blood pressure, diastolic blood pressure, mean arterial pressure, plasma renin and aldosterone. Figure S5. Effects of salt supplements on body weight, eGFR, and albuminuria. Figure S6. Effects of salt supplements on plasma magnesium, calcium, and phosphate. Figure S7. Effects of salt supplements on endothelin-1 and urine cortisol to cortisone ratio. Table S1. Baseline characteristics. Table S2. Characteristics of patients developing hyperkalemia. Table S3. Comparison of baseline characteristics between patients who did or did not develop hyperkalemia during one of the treatments. Table S4. Subanalysis comparing plasma bicarbonate and potassium in participants with or without thiazide diuretic use. CONSORT Checklist. [file mmc1.docx]

**Supplementary Material**

**Randomized cross-over trial of electrolyte, acid-base**

**and blood pressure effects of salt supplements in chronic kidney disease**

Michiel L.A.J. Wieërs, Usha Musterd-Bhaggoe, Yoëlle Goos, Ingrid M. Garrelds,

Sjoerd van den Berg, A.H. Jan Danser, Lynda Frassetto, Martin H. de Borst, Liffert Vogt,

Joris Rotmans, Pedro H. Imenez Silva, Ewout J. Hoorn

**Table of contents**

- Supplementary Methods
- **Table S1.** Baseline characteristics
- **Table S2.** Characteristics of patients developing hyperkalemia
- **Table S3.** Comparison of baseline characteristics between patients who did or did not develop hyperkalemia during one of the treatments.
- **Table S4.** Subanalysis comparing plasma bicarbonate and potassium in participants with or without thiazide diuretic use.
- **Figure S1.** CONSORT diagram.
- **Figure S2.** Effects of salt supplements on net acid excretion.
- **Figure S3.** Flowchart for management of hyperkalemia.
- **Figure S4.** Effects of supplements on home systolic blood pressure, diastolic blood pressure, mean arterial pressure, plasma renin and aldosterone.
- **Figure S5.** Effects of salt supplements on body weight, eGFR, and albuminuria
- **Figure S6.** Effects of salt supplements on plasma magnesium, calcium, and phosphate
- **Figure S7.** Effects of salt supplements on endothelin-1 and urine cortisol to cortisone ratio
- Supplemental references
- CONSORT Checklist

**Supplementary Methods**

***General overview***

Thirty-one patients with CKD stage G3b or G4 who were using renin–angiotensin inhibitors were included in a placebo-controlled, double-blind, randomized cross-over trial. The trial lasted six weeks and was divided into randomized treatment blocks consisting of placebo, potassium chloride, sodium chloride, potassium bicarbonate, sodium bicarbonate, and potassium gluconate (**Figure S1**). Each treatment phase lasted until the study visit of the 5^th^ day and was followed by a two-day washout period. The oral salt supplements provided 40 mmol/day of potassium or sodium. The primary endpoint was plasma bicarbonate. Potassium gluconate was selected as acid-base neutral potassium salt.

***Study design***

The study was a randomized, double-blind, placebo-controlled, single-center crossover trial with five potassium and sodium salts (**Figure S1**). The study was approved by the institutional review board of the Erasmus Medical Center (MEC-2023-0510) and registered at ClinicalTrials.gov (NCT06237712). An independent statistician generated the random allocation sequence using a random number generator, employing simple randomization. The research team did not have access to the sequence. The statistician provided the randomization list to the laboratory responsible for producing the supplements (see below). The laboratory prepared one box per participant, containing the five supplements and the placebo in the order specified by the randomization list. This procedure ensured that both participants and the research team remained blinded. All study visits were conducted at the Erasmus Medical Center in Rotterdam, The Netherlands. Inclusion criteria were: age ≥ 18 years, an estimated glomerular filtration rate (eGFR) of 15-44 ml/min/1.73 m² (calculated using the 2009 CKD-EPI equation without the Black race coefficient^S13^), and the use of renin-angiotensin inhibitors. Exclusion criteria included the use of potassium-sparing diuretics, potassium binders, mineralocorticoid receptor antagonists, sodium bicarbonate supplements, and simultaneous use of both angiotensin-converting enzyme-inhibitors and angiotensin receptor blockers. Additionally, patients with a history of kidney transplantation, current use of immunosuppressive drugs, chronic respiratory acidosis or hyperkalemia (plasma potassium >5.5 mmol/L) were excluded. Patients were recruited between 26 January 2024 and 21 March 2025. No important changes were made to the trial after it started. Patients continued to receive their normal outpatient care during participation in the trial.

***Outcome parameters and power calculation***

The primary outcome was plasma bicarbonate. Secondary outcomes included other acid-base parameters, as well as electrolyte levels in blood and urine, and blood pressure. Power calculations were based on a 6-treatment cross-over design analyzed using a linear mixed-effects model with fixed effects for treatment and period and a random subject effect. Assuming a within-subject standard deviation of 0.9 units and a clinically meaningful treatment–control difference of 0.8 units, 26 evaluable subjects provide 80% power using a two-sided α of 0.05 adjusted for five treatment comparisons. Allowing for approximately 15% dropout across six periods, we decided to enroll 31 subjects.

***Supplements***

The supplements were prepared as visually indistinguishable capsules by Laboratorium Medisan (Heerenveen, The Netherlands). In a randomized order, participants received placebo (potato starch) or daily doses of 40 mmol of one of the following supplements: potassium chloride, sodium chloride, potassium bicarbonate, sodium bicarbonate, or potassium gluconate. Each participant was allocated a box at the trial center containing their bottles with supplements, which were arranged in a randomized order and labeled only with numbers 1/6 to 6/6. Participants were instructed to take 3 x 3 capsules per day during meals from Monday until the study visit on Friday. The remainder of the Friday and the weekend served as a wash-out period. On the following Monday, participants resumed the trial with the next supplement or placebo. During the trial, participants were instructed to maintain their usual diets.

***Measurements***

For each study visit, 24-hour urine was collected (from 8:00 AM the day before until 8:00 AM on the day of the visit and preserved under mineral oil), alongside venous blood and spot urine samples. Blood pressure was measured at home throughout the week (9 measurements/day, including 3 measurements in the morning, afternoon and evening) using an automated device (Wireless Blood Pressure Monitor, Andon Health, Tianjin, China). Blood potassium, pH, and bicarbonate were analyzed with a blood gas analyzer (ABL90 Series, Radiometer, Copenhagen, Denmark), while all other electrolytes in blood and urine were measured on an automated Cobas 8000 platform (Roche Diagnostics, Basel, Switzerland). Plasma renin and aldosterone were quantified by radioimmunoassay (Immunotech, Prague, Czech Republic), endothelin-1 by ELISA (Quantikine, R&D Systems, Minneapolis, USA), urine ammonium using the Berthelot method^S14^, citrate with the Megazyme Citric Acid Assay Kit (Megazyme, Bray, Ireland), and urine pH with a pH meter (HI15221, Hanna Instruments, Nieuwegein, The Netherlands). Titratable acidity was calculated using an equation with urine phosphate, creatinine and pH.^S15^ Net acid excretion was calculated as (urine ammonium + titratable acidity) – urine bicarbonate.

Finally, urine cortisol and cortisone were measured by liquid chromatography-mass spectrometry^.S16^

***Safety***

Serious adverse events were defined as untoward medical occurrences or effects that resulted in death or requiring hospitalization for electrolyte disorders, cardiac problems or renal problems. Adverse events such as hematoma after blood draw or gastro-intestinal discomfort were evaluated at each study visit. Patients were instructed to call the study team if their home blood pressure exceeded 160/90 mmHg for an entire day. Patients whose blood potassium levels exceeded 5.5 mmol/L were managed according to a flowchart (**Figure S2**). Specific criteria for withdrawal from the study were persistent hyperkalemia, inability to tolerate supplements, and hypertension unresponsive to escape medication. No interim analysis was performed.

***Statistics***

Data were tested for normal distribution using the Shapiro-Wilks test. Normally distributed data were expressed as mean ± standard deviation. Non-normally distributed data were expressed as median with interquartile range (IQR). Primary and secondary outcomes were analyzed using a linear-mixed effect model with the placebo-period as comparator. The effect of time was tested and no effect was identified for the primary and secondary outcomes. Unpaired t-tests were used to compare patients with and without hyperkalemia and those with and without thiazide diuretic use. All randomized participants were included in the analysis. The maximum percentage of missing data was 4% and therefore data were not imputed. The data were analyzed using R (R Core Team, 2023) and GraphPad (Version 10.6.1, Boston, MA, USA). An adjusted p-value < 0.05 was considered statistically significant.

**Table S1.** Baseline characteristics.

|  | **Characteristics** | **All participants**  **(n = 31)** |
| --- | --- | --- |
| **General data** | Age, years | 69 ± 11 |
|  | Female sex, n (%) | 9 (29) |
|  | Body mass index, kg/m^2^ | 28.5 ± 4.8 |
| **Self-reported ethnicity** | Black | 1 (3) |
|  | Mixed | 3 (10) |
|  | White | 27 (87) |
| **Office blood pressure** | Systolic blood pressure, mmHg | 135 ± 18 |
|  | Diastolic blood pressure, mmHg | 79 ± 10 |
| **Cause of CKD** | Diabetic kidney disease | 12 (39) |
|  | Hypertensive/vascular disease | 9 (29) |
|  | Other causes* | 10 (32) |
| **Comorbidity** | Diabetes, n (%) | 12 (39) |
|  | Hypertension, n (%) | 21 (68) |
| **Laboratory parameters** | eGFR, ml/min per 1.73m^2^ | 29 ± 7 |
|  | Baseline plasma potassium, mmol/L | 4.6 ± 0.5 |
|  | Baseline plasma bicarbonate, mmol/L | 25 ± 3 |
| **Medication** | Angiotensin receptor blockers, n (%) | 16 (52) |
|  | ACE-inhibitors, n (%) | 15 (48) |
|  | Calcium channel blockers, n (%) | 19 (61) |
|  | Thiazide diuretics, n (%) | 9 (29) |
|  | Loop diuretics, n (%) | 1 (3) |
|  | SGLT-2 inhibitors, n (%) | 4 (13) |
|  | Alpha blockers, n (%) | 3 (10) |

**Footnote:** *Includes inherited causes, glomerular diseases, and unknown causes.

**Table S2.** Characteristics of patients developing hyperkalemia

| **Patient ID** | **Age** | **Sex** | **eGFR** | **Supplement** | **Potassium on supplement** | **Potassium at baseline** |
| --- | --- | --- | --- | --- | --- | --- |
| 3 | 61 | Male | 22 | KGluconate | 5.6 | 5.2 |
| 7 | 77 | Female | 16 | KGluconate | 5.9 | 5.3 |
|  |  |  |  | KCl | 6.0 |  |
|  |  |  |  | NaHCO3 | 6.0 |  |
| 9 | 76 | Male | 21 | KHCO3 | 5.7 | 5.5 |
| 11 | 83 | Male | 31 | KCl | 6.1* | 4.5 |
| 26 | 50 | Female | 19 | KCl | 5.6 | 5.0 |
|  |  |  |  | KHCO3 | 6.2* |  |
| 29 | 61 | Male | 22 | KGluconate | 5.6 | 5.0 |

**Footnote:** *In these patients an ECG was performed, but did not show signs of hyperkalemia. See also **Figure S3**.

**Table S3.** Comparison of baseline characteristics between patients who did or did not develop hyperkalemia during one of the treatments.

|  | **No hyperkalemia**  (n = 25) | **Hyperkalemia**  (n = 6) | **P-value** |
| --- | --- | --- | --- |
| Age, years | 69 ± 11 | 69 ± 12 | 0.9 |
| eGFR, ml/min per 1.73 m^2^ | 30 ± 7 | 22 ± 4 | 0.003 |
| Baseline plasma potassium, mmol/L | 4.5 ± 0.5 | 5.1 ± 0.3 | 0.005 |

**Table S4.** Subanalysis comparing plasma bicarbonate and potassium in participants with or without thiazide diuretic use.

| **Blood parameter** | **Treatment arm** | **Non-thiazide users (n = 22)** | **Thiazide users (n = 9)** | **P-value** |
| --- | --- | --- | --- | --- |
| **Plasma bicarbonate** | Baseline | 24.8 ± 2.6 | 25.7 ± 4.0 | 0.4 |
|  | Placebo | 24.2 ± 3.0 | 26.4 ± 2.8 | 0.08 |
|  | KCl | 24.5 ± 2.9 | 24.5 ± 3.0 | 1.0 |
|  | NaCl | 24.6 ± 3.0 | 26.4 ± 3.1 | 0.1 |
|  | KBic | 26.9 ± 2.7 | 28.0 ± 2.1 | 0.3 |
|  | NaBic | 27.5 ± 2.3 | 29.3 ± 2.9 | 0.08 |
|  | KGlc | 26.2 ± 2.9 | 26.9 ± 3.9 | 0.6 |
| **Plasma potassium** | Baseline | 4.7 ± 0.5 | 4.4 ± 0.6 | 0.3 |
|  | Placebo | 4.5 ± 0.3 | 4.2 ± 0.6 | 0.07 |
|  | KCl | 4.9 ± 0.5 | 4.9 ± 0.9 | 0.9 |
|  | NaCl | 4.4 ± 0.3 | 4.2 ± 0.5 | 0.1 |
|  | KBic | 4.8 ± 0.6 | 4.6 ± 0.6 | 0.5 |
|  | NaBic | 4.4 ± 0.4 | 4.3 ± 0.8 | 0.6 |
|  | KGlc | 4.9 ± 0.5 | 4.6 ± 0.7 | 0.1 |

**Figure S1.** CONSORT diagram.

**
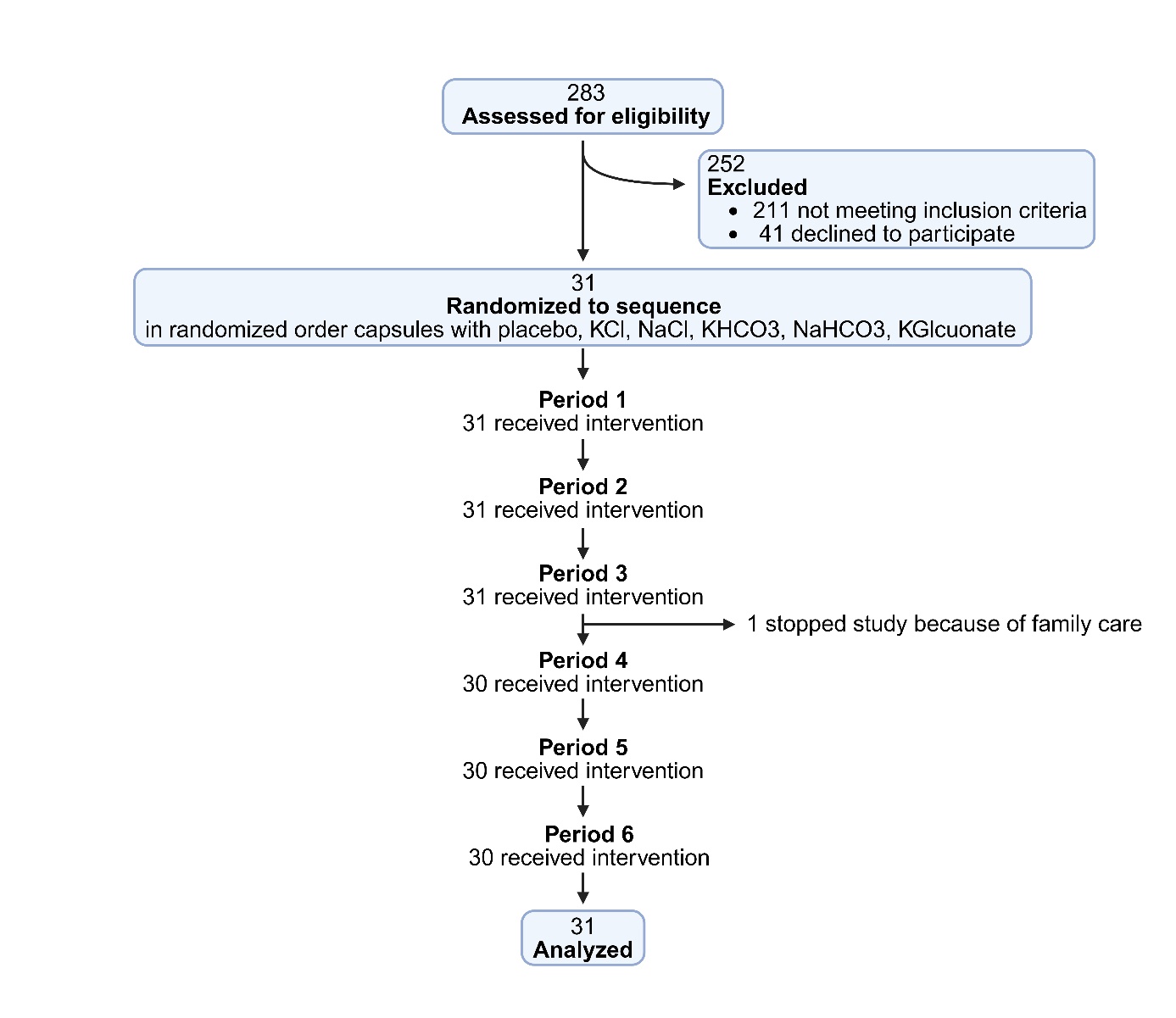
**

**Legend:** CONSORT diagram for the study. Participants attended seven weekly study visits, including one baseline visit and six intervention visits (Periods 1-6). Study supplements were given on weekdays and contained either 40 mmol sodium or potassium per day. The weekend was used for wash-out.

**Figure S2.** Effects of salt supplements on net acid excretion


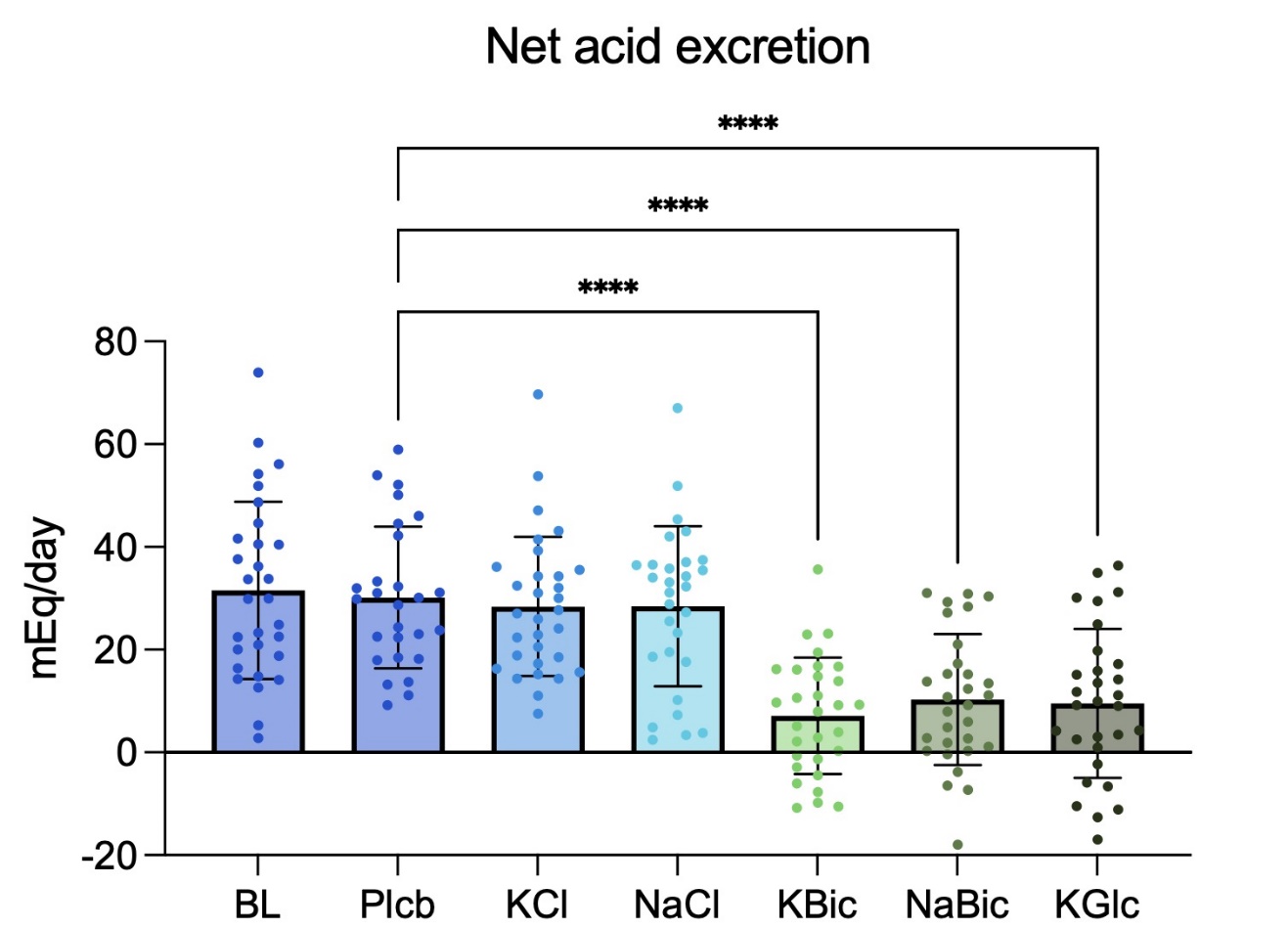


Data were analyzed using a linear mixed-effects model with subject included as a random intercept and treatment and period included as fixed effects. Post-hoc comparisons were adjusted for multiple testing using Tukey’s test.

**Abbreviations:** BL, baseline; Plcb, placebo; KCl, potassium chloride; NaCl, sodium chloride; KBic, potassium bicarbonate; NaBic, sodium bicarbonate; KGlc, potassium gluconate. **Adjusted p-value:** **** P ≤ 0.0001

**Figure S3.** Flowchart for management of hyperkalemia.


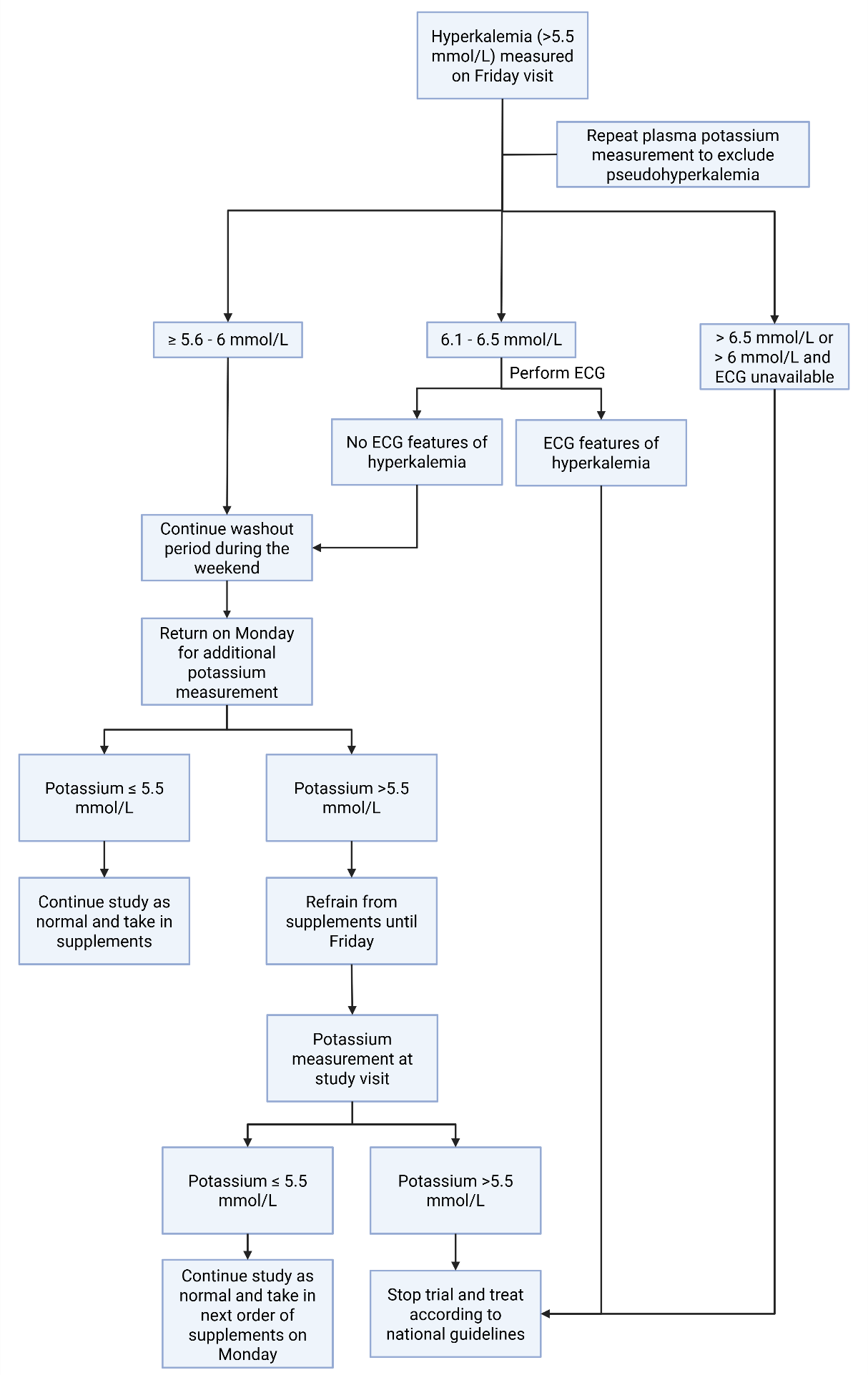


**Figure S4.** Effects of supplements on home systolic blood pressure, diastolic blood pressure, mean arterial pressure, plasma renin and aldosterone


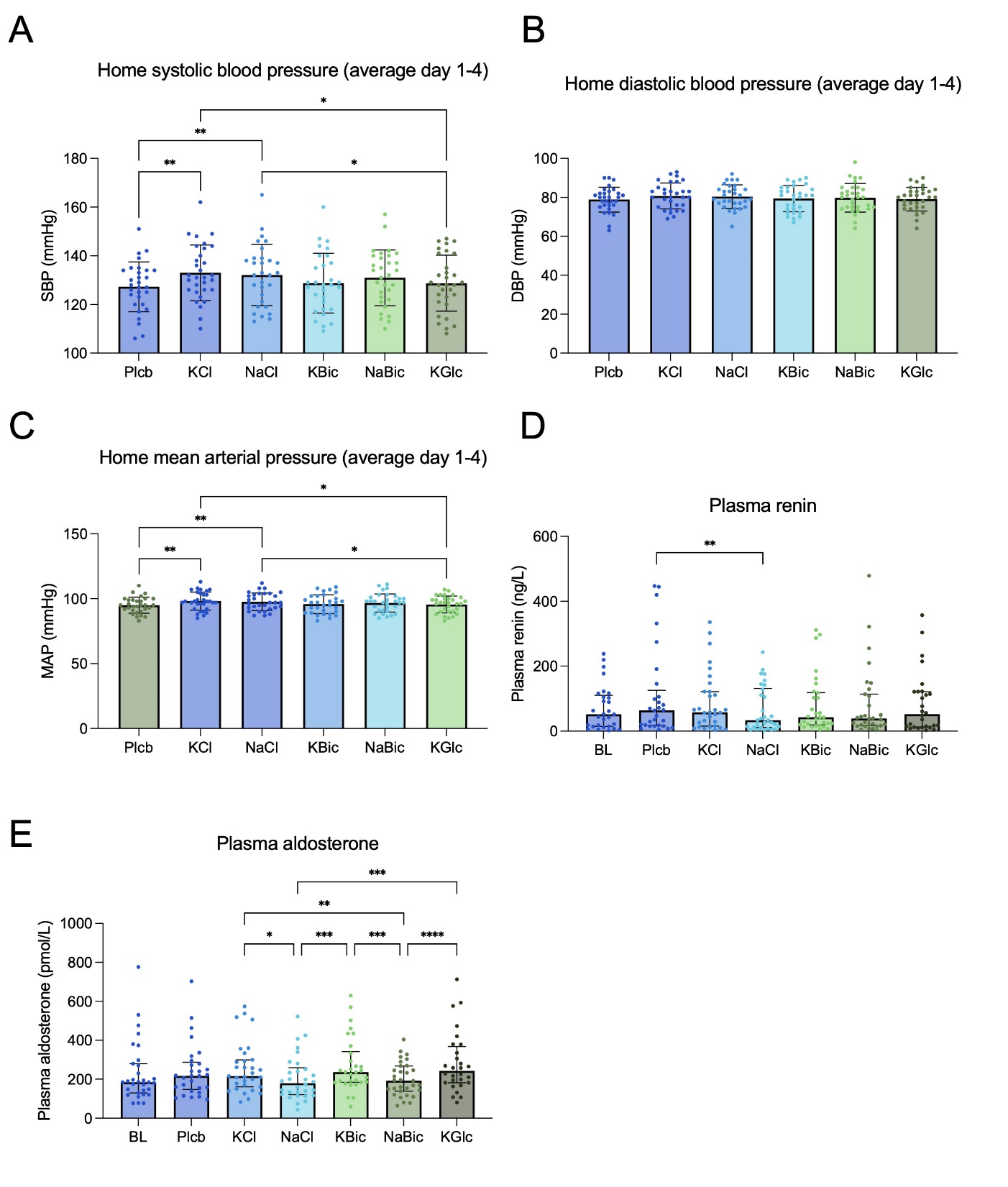
 Data were analyzed using a linear mixed-effects model with subject included as a random intercept and treatment and period included as fixed effects. Post-hoc comparisons were adjusted for multiple testing using Tukey’s test.

**Abbreviations:** BL, baseline; Plcb, placebo; KCl, potassium chloride; NaCl, sodium chloride; KBic, potassium bicarbonate; NaBic, sodium bicarbonate; KGlc, potassium gluconate. **Adjusted p-values:** * P ≤ 0.05, ** P ≤ 0.01, *** P ≤ 0.001. **** P ≤ 0.0001

**Figure S5.** Effects of salt supplements on body weight, eGFR, and albuminuria

**
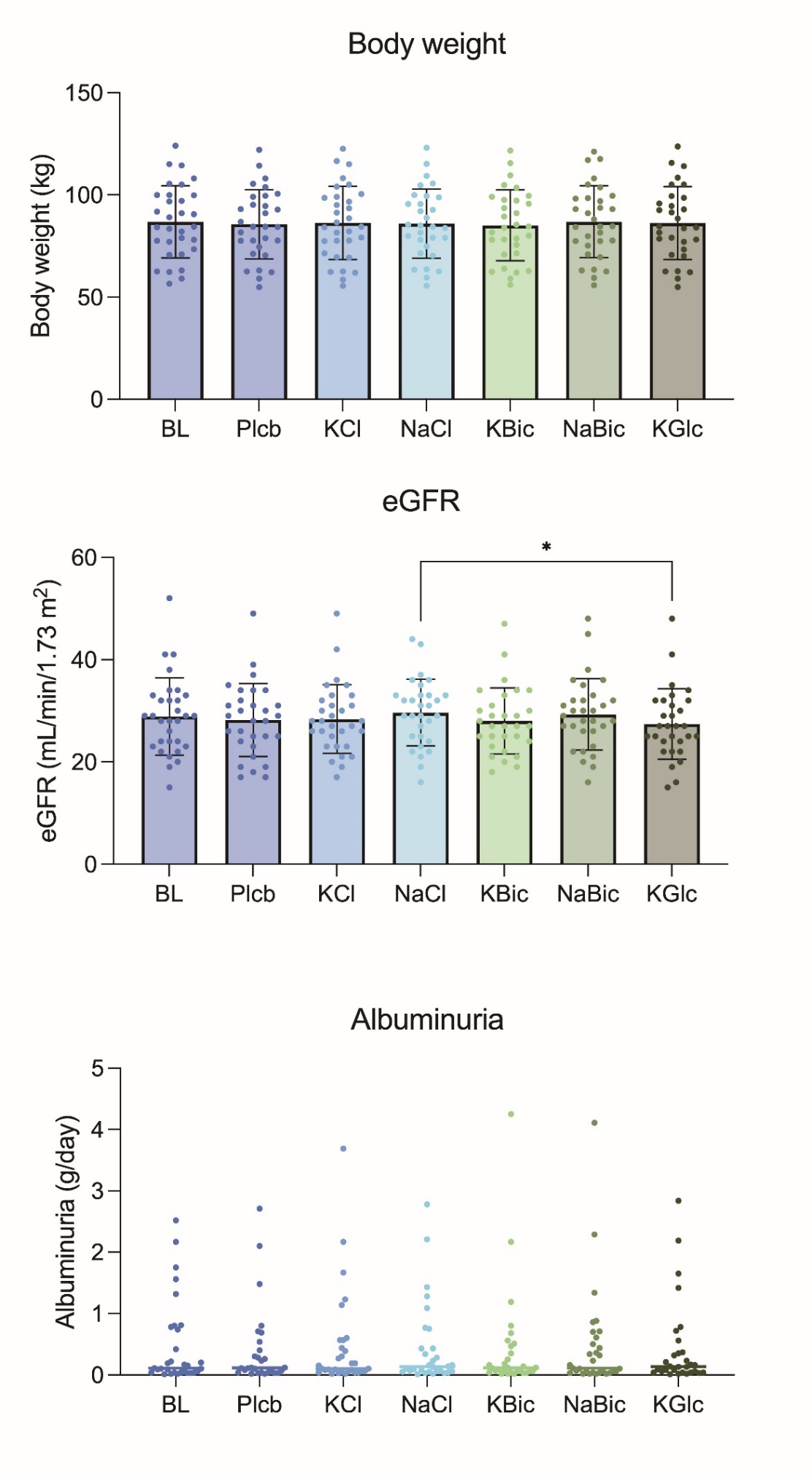
**

Data were analyzed using a linear mixed-effects model with subject included as a random intercept and treatment and period included as fixed effects. Post-hoc comparisons were adjusted for multiple testing using Tukey’s test.

**Abbreviations:** BL, baseline; Plcb, placebo; KCl, potassium chloride; NaCl, sodium chloride; KBic, potassium bicarbonate; NaBic, sodium bicarbonate; KGlc, potassium gluconate.

**Adjusted p-value:** * P ≤ 0.05

**Figure S6.** Effects of salt supplements on plasma magnesium, calcium, and phosphate


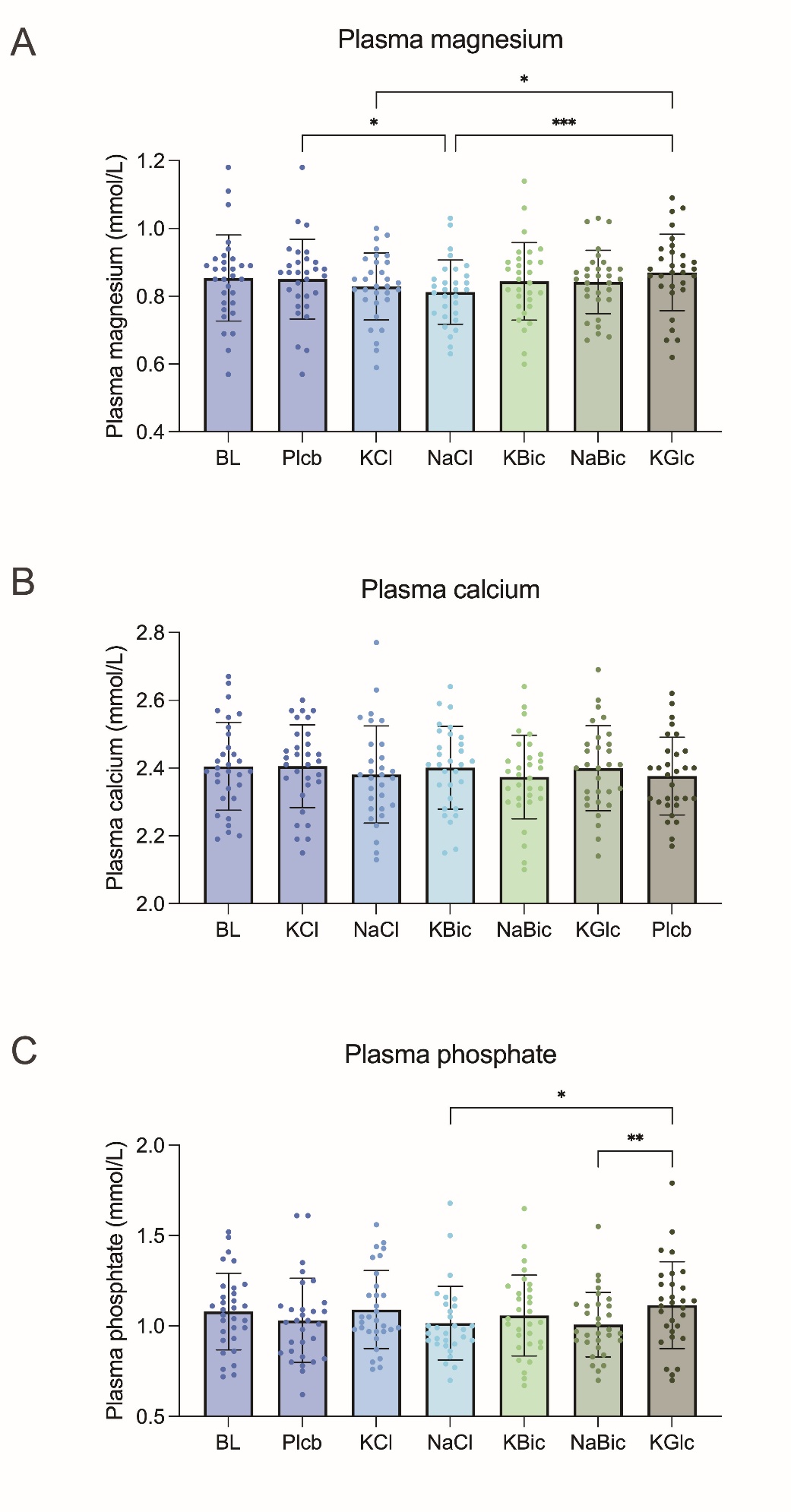


Data were analyzed using a linear mixed-effects model with subject included as a random intercept and treatment and period included as fixed effects. Post-hoc comparisons were adjusted for multiple testing using Tukey’s test.

**Abbreviations:** BL, baseline; Plcb, placebo; KCl, potassium chloride; NaCl, sodium chloride; KBic, potassium bicarbonate; NaBic, sodium bicarbonate; KGlc, potassium gluconate. **Adjusted p-values:** * P ≤ 0.05, ** P ≤ 0.01, *** P ≤ 0.001

**Figure S7.** Effects of salt supplements on endothelin-1 and urine cortisol to cortison ratio


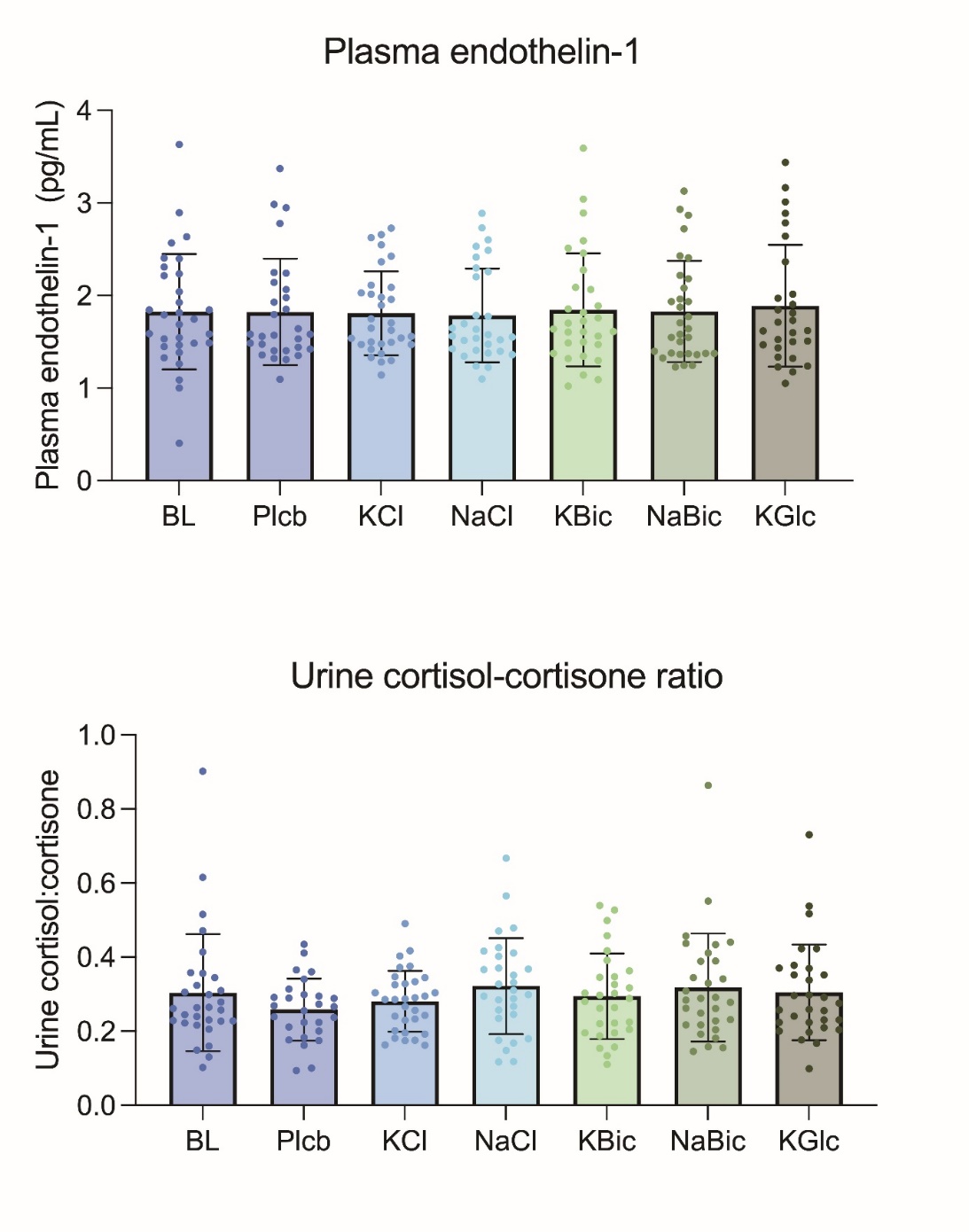


Data were analyzed using a linear mixed-effects model with subject included as a random intercept and treatment and period included as fixed effects. Post-hoc comparisons were adjusted for multiple testing using Tukey’s test.

**Abbreviations:** BL, baseline; Plcb, placebo; KCl, potassium chloride; NaCl, sodium chloride; KBic, potassium bicarbonate; NaBic, sodium bicarbonate; KGlc, potassium gluconate.

**Supplemental references**

S1. Naylor JM, Forsyth GW. The alkalinizing effects of metabolizable bases in the healthy calf. *Can J Vet Res* 1986; **50:** 509-516.

S2. Stone MS, Martin BR, Weaver CM. Short-Term Supplemental Dietary Potassium from Potato and Potassium Gluconate: Effect on Calcium Retention and Urinary pH in Pre-Hypertensive-to-Hypertensive Adults. *Nutrients* 2021; **13**.

S3. Carr AJ, Slater GJ, Gore CJ*, et al.* Effect of sodium bicarbonate on [HCO3-], pH, and gastrointestinal symptoms. *Int J Sport Nutr Exerc Metab* 2011; **21:** 189-194.

S4. Filippini T, Naska A, Kasdagli MI*, et al.* Potassium Intake and Blood Pressure: A Dose-Response Meta-Analysis of Randomized Controlled Trials. *J Am Heart Assoc* 2020; **9:** e015719.

S5. Gritter M, Rotmans JI, Hoorn EJ. Role of Dietary K(+) in Natriuresis, Blood Pressure Reduction, Cardiovascular Protection, and Renoprotection. *Hypertension* 2019; **73:** 15-23.

S6. Wouda RD, Karsten M, Michels EHA*, et al.* Natriuretic Response to an Acute Oral Potassium Load in Healthy Individuals and Patients with Chronic Kidney Disease: A Randomized Controlled Trial. *Nephrol Dial Transplant* 2026; **41:** 255-264.

S7. Boyd-Shiwarski CR, Weaver CJ, Beacham RT*, et al.* Effects of extreme potassium stress on blood pressure and renal tubular sodium transport. *Am J Physiol Renal Physiol* 2020; **318:** F1341-F1356.

S8. Gritter M, Wei KY, Wouda RD*, et al.* Chronic kidney disease increases the susceptibility to negative effects of low and high potassium intake. *Nephrol Dial Transplant* 2024; **39:** 795-807.

S9. Ibrahim HN, Hostetter TH. Role of dietary potassium in the hyperaldosteronism and hypertension of the remnant kidney model. *J Am Soc Nephrol* 2000; **11:** 625-631.

S10. Rabelink TJ, Koomans HA, Hene RJ, Dorhout Mees EJ. Early and late adjustment to potassium loading in humans. *Kidney Int* 1990; **38:** 942-947.

S11. Wu A, Wolley MJ, Mayr HL, *et al*. Randomized Trial on the Effect of Oral Potassium Chloride Supplementation on the Thiazide-Sensitive Sodium Chloride Cotransporter in Healthy Adults. *Kidney Int Rep* 2023; **8:** 1201-1212.

S12. Shore AC, Markandu ND, MacGregor GA. A randomized crossover study to compare the blood pressure response to sodium loading with and without chloride in patients with essential hypertension.  *J Hypertens* 1988; **6:** 613-7

S13. Gansevoort RT, Anders HJ, Cozzolino M*, et al.* What should European nephrology do with the new CKD-EPI equation? *Nephrol Dial Transplant* 2023; **38:** 1-6.

S14. Cunarro JA, Weiner MW. A comparison of methods for measuring urinary ammonium. *Kidney Int* 1974; **5:** 303-305.

S15. Kok DJ, Poindexter J, Pak CY. Calculation of titratable acidity from urinary stone risk factors. *Kidney Int* 1993; **44:** 120-6.

S16. Mirzaian M, van Zundert SKM, Schilleman WF*, et al.* Determination of cortisone and cortisol in human scalp hair using an improved LC-MS/MS-based method. *Clin Chem Lab Med* 2024; **62:** 118-127.
